# REGIONAL NEUROANATOMIC EFFECTS ON BRAIN AGE INFERRED USING MAGNETIC RESONANCE IMAGING AND RIDGE REGRESSION

**DOI:** 10.1093/geroni/igae098.0566

**Published:** 2024-12-31

**Authors:** Andrei Irimia

**Affiliations:** University of Southern California, Los Angeles, California, United States

## Abstract

TBD

